# Clinical Vignette of Bacterial Meningitis Complicated by a Cerebrovascular Event

**DOI:** 10.7759/cureus.30048

**Published:** 2022-10-07

**Authors:** Abhishek S Bhutada, Thomas V Kodankandath

**Affiliations:** 1 Neurosurgery, Virginia Tech Carilion School of Medicine, Roanoke, USA; 2 Neurology, Virginia Tech Carilion School of Medicine, Roanoke, USA

**Keywords:** infectious disease pathology, cardiovascular complications, neurologic injury, embolic cva, pneumococcal meningitis

## Abstract

Community-acquired bacterial meningitis is a life-threatening illness that is commonly caused by *Streptococcus pneumoniae*. Often, it can be associated with high mortality and morbidity rates especially due to the frequency of added neurological complications like hydrocephalus, seizures, stroke, and cerebrovascular events. Here we present an unusual case of cerebrovascular infarction complicating the outcome of a patient who suffered from community-acquired bacterial meningitis.

## Introduction

In the United States nearly 4,000 cases of bacterial meningitis present to the emergency department annually with *Streptococcus pneumoniae* representing approximately 51% of the cases [1,2]. The bacterial invasion via bacteremia and resultant inflammation of the subarachnoid matter is a life-threatening medical emergency that requires prompt diagnosis and treatment. Cerebrovascular events are an associated neurological complication related to bacterial meningitis.

The case we have outlined here displays some of the complications that can arise in the setting of bacterial meningitis. We offer critical insights on some of the detection and diagnostic measures in the setting of cerebrovascular events that can complicate bacterial meningitis pathogenesis.

## Case presentation

A 55-year-old female who runs a daycare facility presents to the emergency room due to four days of worsening left knee pain and left shoulder-to-back pain with associated numbness and tingling. Her situation is complicated by flu-like symptoms of fever, headache, nausea, vomiting, and worsening mental status, which is noted by increased lethargy, decreased responsiveness, and slurred speech as noted by her family. She was brought to the ED for further evaluation. The patient denied any sick contacts or recent travel. The patient was not on any immunosuppressive medications.

Examination revealed a temperature of 100.2 F, poorly responsive, somnolent, and not following commands (Glasgow Coma Scale 11) with notable nuchal rigidity, hyperreflexive 3/4 in the biceps, patellar, and Achilles. The complete blood count was remarkable for an elevated white blood count with elevated segmented cells (see Table 1). With a high clinical suspicion, a lumbar puncture was performed, and cloudy cerebrospinal fluid was remarkable for elevated white blood cells with a neutrophil predominance (see Table 1). Gram stain was significant for gram-positive cocci in pairs that eventually grew *S. pneumoniae*. The patient was initially treated with Vancomycin, Ceftriaxone, rifampin, and adjunctive dexamethasone. As sensitivities became available, she was changed to Penicillin-G and completed a total of 14 days of antibiotic treatment.

**Table 1:** Abnormal lab values

Lab Study Type	Variable	Lab Value	Reference Range
CBC	WBC	18.5 (x10^3^ per μL)	4.5-11 (x10^3^ per μL)
	Segmented Cells	89%	40%-60%
CSF	WBC	5,890 leukocytes/mm	0-8 leukocytes/mm

As her mental status improved, a physical exam revealed normal speech, cranial nerves were intact with the exception of right lateral rectus palsy, reflexes were 3/4 in lower extremities with equivocal plantar reflexes, strength was 4/5 on the left side with a 3/5 left-hand grip strength and decreased range of motion on left lower extremity. No muscle wasting was noted. An MRI without contrast identified an acute infarct located in the right parietal lobe. Inpatient telemetry monitoring and transthoracic echocardiogram did not show any evidence of a cardioembolic source. Her CVA was managed conservatively, and her symptoms of weakness improved to baseline with physical therapy. Her lateral rectus palsy persisted and as a result, she complained of diplopia. She was followed up in the ambulatory clinic by Neurology and Ophthalmology for her residual symptoms.

## Discussion

To understand the pathophysiology of cerebrovascular complications, it is important to review the physiology of cerebral blood flow. An autoregulatory mechanism is responsible for maintaining the relatively constant cerebral blood flow. Autoregulation modulates cerebrovascular resistance based on the metabolic needs of the brain. For example, during seizures or fever, the increased metabolic demand is met by increasing the vasodilatory properties of cerebral arteries. A low arterial PaO_2_ (<50mmHg) and high PaCO_2_ have vasodilatory effects on cerebral circulation. Alternatively, hypocapnia reduces blood flow in the cerebral vasculature. For this reason, hyperventilation can be used in the setting of increased intracranial pressure [3].

An increase in intracranial pressure can occur in patients with cerebrovascular complications of bacterial meningitis. Increased ICP can occur due to vasogenic edema resulting from endothelial damage, cytotoxic edema related to infarct of the brain parenchyma, or increased intracranial blood volume, as occurs in sinus venous thrombosis. This increase in ICP leads to decreased cerebral perfusion pressure (MAP-ICP); thereby, predisposing the necrosis of the brain parenchymal tissues, especially cortical regions [4]. Headache, confusion, irritability, nausea, and vomiting are seen in mild cases of increased ICP. However, more severe cases of increased ICP can result in coma, hypotension or hypertension (Cushing reflex), papilledema, cranial nerve palsy especially CN VI, and death from the increased risk of brain herniations [5].

The cerebrovascular complications can be divided into pathology at the level of large arteries at the base of the brain, medium-sized arteries, small pial and intraparenchymal vessels, and major sinuses and cortical veins. The large arterial narrowing often occurs at the supraclinoid portion of the internal carotid artery. At this site, heavy subarachnoid exudate from the inflammatory processes of meningitis in the cisterns and the subarachnoid spaces bathes the internal carotid, basilar, and vertebral arteries and their primary branches. The subarachnoid purulent exudate influences cerebral vessel changes secondary to exudative infiltration to the vessel and vascular changes resulting from the surrounding inflammation. It can also cause a vasospastic response to the infection. The medium-sized arteries are the second group of vessels that may be affected by the inflammatory complications of meningitis. Mainly the leptomeningeal arterial branches are affected directly due to meningitis or due to an embolism from concurrent infective endocarditis. The third type of cerebrovascular complication affects the small pial and intraparenchymal vessels [4]. These vessels are also susceptible to polymorphonuclear infiltration to their location in the subintimal layer as shown by Dodge et al. [6]. Angiographically, this can be evident through a hypervascular pattern, which can appear as a blush resulting from the dilations of the small arterioles and capillaries [7,8]. This focal hyperperfusion could be explained by loss of cerebral autoregulation, decrease in extracellular space pH, or vasodilatory mediators from the inflammatory process. Finally, the major sinuses and cortical veins are also susceptible to inflammatory involvement secondary to their thin collagenous wall, sparse muscle cells, and elastic tissue. Relative slow blood flow in the veins compared to arteries makes them susceptible to inflammatory cell adherence and vasculitis [4].

Corticosteroids can reduce intracranial pressure, brain edema formation, and the inflammatory response from meningitis. Therefore, improved CSF hydrodynamics and cerebral blood flow can prevent cerebrovascular complications seen in meningitis. Anticoagulation with heparin is not recommended in cases with septic sinus venous thrombosis in bacterial meningitis secondary to an increased risk of venous hemorrhagic infarctions.

Despite the development of antibiotics in bacterial meningitis, the mortality and sequelae remain high. To combat this the approach must be directed toward early detection and intervention of the complications as seen in the disease process [4]. Kelin et al. have shown that increased cerebral blood flow velocities (CBFv) using transcranial Doppler (TCD) was associated with approximately 10 times the risk of having ischemic stroke and unfavorable outcome. Studies show that TCD is a very easy and useful investigational tool that can be done at the bedside to detect patients with a high risk of having a stroke and unfavorable outcomes [9].

## Conclusions

Understanding the pathophysiology of cerebrovascular events in the setting of bacterial meningitis is a critical component of diagnosis and treatment. Although these events are uncommon in the setting of bacterial meningitis, they can cause severe complications and a poor prognosis for the patient. Early detection of these events can improve the prognosis. Through this report, we hope to highlight some of the key clinical features one might encounter in the setting of bacterial meningitis with cerebrovascular complications to improve diagnosis and hopefully promote better outcomes.
